# Advancing renewable fuel integration: A comprehensive response surface methodology approach for internal combustion engine performance and emissions optimization

**DOI:** 10.1016/j.heliyon.2023.e22238

**Published:** 2023-11-11

**Authors:** Johnny Koh Siaw Paw, Tiong Sieh Kiong, Mohd Kamal Kamarulzaman, Abdullah Adam, Sakinah Hisham, K. Kadirgama, D. Ramasamy, Chong Tak Yaw, Ahmad Fitri Yusop, Talal Yusaf, Hayder A. Dhahad, F. Benedict

**Affiliations:** a Institute of Sustainable Energy, University Tenaga Nasional, Putrajaya Campus, 43000 Kajang, Malaysia; b Advanced Nano Coolant-Lubricant (ANCL) Lab, Automotive Engineering Centre, Universiti Malaysia Pahang, Pekan 26600, Pahang, Malaysia; c Faculty of Mechanical and Automotive Engineering Technology, Universiti Malaysia Pahang, Pekan 26600, Pahang, Malaysia; d Department of Civil Engineering, College of Engineering, Almaaqal University, Basra, 61003, Iraq; e School of Engineering and Technology, Central Queensland University, Brisbane, QLD 4008, Australia; f Mechanical Engineering Department, University of Technology, Baghdad 19006, Iraq; g Enhanced Track, No. 9, Jalan Meranti Jaya 12, Meranti Jaya Industrial Park, Puchong 47120, Malaysia

**Keywords:** Energy, Design of experiments, Response surface methodology, Internal combustion engine, Alternative fuel

## Abstract

In the realm of internal combustion engines, there is a growing utilization of alternative renewable fuels as substitutes for traditional diesel and gasoline. This surge in demand is driven by the imperative to diminish fuel consumption and adhere to stringent regulations concerning engine emissions. Sole reliance on experimental analysis is inadequate to effectively address combustion, performance, and emission issues in engines. Consequently, the integration of engine modelling, grounded in machine learning methodologies and statistical data through the response surface method (RSM), has become increasingly significant for enhanced analytical outcomes. This study aims to explore the contemporary applications of RSM in assessing the feasibility of a wide range of renewable alternative fuels for internal combustion engines. Initially, the study outlines the fundamental principles and procedural steps of RSM, offering readers an introduction to this multifaceted statistical technique. Subsequently, the study delves into a comprehensive examination of the recent applications of alternative renewable fuels, focusing on their impact on combustion, performance, and emissions in the domain of internal combustion engines. Furthermore, the study sheds light on the advantages and limitations of employing RSM, and discusses the potential of combining RSM with other modelling techniques to optimise results. The overarching objective is to provide a thorough insight into the role and efficacy of RSM in the evaluation of renewable alternative fuels, thereby contributing to the ongoing discourse in the field of internal combustion engines.

## Highlights

• A systematic review of RSM and the internal combustion engine is presented.
• The theoretical principles of RSM and steps for its application are summarized.
• Overview of current research on the application of RSM in the internal combustion engine.
• The benefits and drawbacks of the RSM are highlighted.
• Efforts to improve RSM by merging with other optimization methods are discussed.

## 1 Introduction

**Fig. 1:**
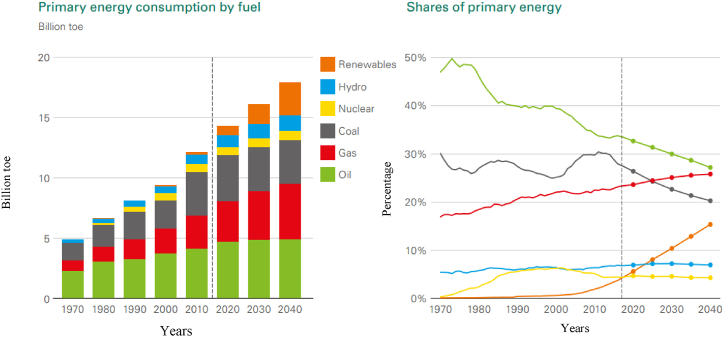
Primary energy consumption and shares of primary world energy [8].

**Fig. 2:**
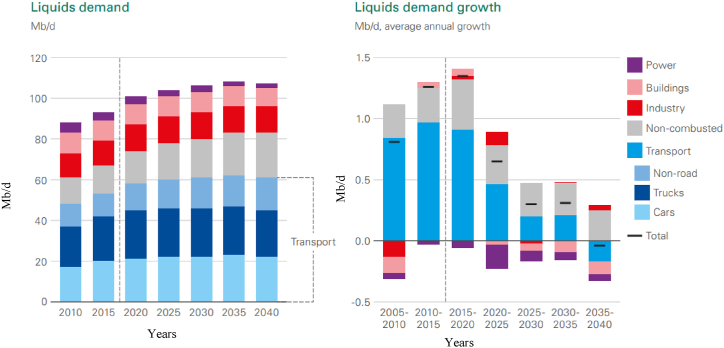
The world demand for liquid fuels [8].

During the period of the Industrial Revolution, spanning the late 18th to the early 19th centuries, there was a marked increase in global energy demand [1]. This sustained demand for energy can be attributed to a significant surge in both population growth and economic development [2,3]. Fig. 1, Fig. 2 project the anticipated demand for energy and liquid fuels through 2040. Petroleum fuel remains a key driver of worldwide economic growth. Currently, there is an ongoing shift towards a lower-carbon energy system, with natural gas and renewable energy sources playing an increasingly pivotal role in energy distribution, surpassing coal and oil. Renewable energy and natural gas are projected to constitute nearly 85 % of primary energy in scenarios of energy transition, with their significance continually outstripping other energy sources. This trend is anticipated to persist into the future [4]. The transportation sector is the primary consumer of liquid fuel, accounting for approximately 55 % of energy usage, and its extensive use has led to environmental challenges, including climate change. The burning of such fuels releases harmful emissions, notably carbon dioxide (CO2), a major contributor to air pollution and detrimental health effects. This issue has garnered global attention, resulting in the implementation of stringent regulations aimed at reducing CO2 emissions [5]. Members of the European Union (EU) have committed to utilising between 10 % and 20 % of green energy for transportation fuel and other energy needs [6]. Additionally, at the Copenhagen Climate Summit in 2009, the United Nations (UN) allocated a budget of US $100 billion to address global climate change challenges [7].

As a solution for energy shortage, alternative renewable fuels have become a potential fuel source globally. The use of alternative renewable fuels is mainly due to their ability to reduce the emissions of engine pollutants in transport vehicles [9]. However, alternative fuels are still under continuous study to improve the accessibility, quality, and feasibility to be compatible with standard fuel performance. Currently, renewable fuels such as liquefied petroleum gas (LPG), compressed natural gas (CNG), hydrogen, biodiesel, and alcohol are possible options to replace standard fuel in spark ignition (SI) and compression ignition (CI) engines [10]. Despite the need for the experimental study to gain a deeper understanding of the engine's characteristics when operating using alternative fuels, lately, there has also been growing attention in using different approaches to evaluate model engine efficiency and exhaust emissions [11]. This modelling is probably among the powerful benefits of minimising operating costs and time by decreasing the dependency on investigational research requirements [12].

The application of response surface methodology (RSM) is quickly becoming an essential and frequently used technique to solve many industrial issues [13]. This approach is often efficient and cost-effective for analysing one and joined factors of experimental variables contributing to output response [14]. In evaluating the literature, it has been revealed that there is a lack of studies that provide the methodologies mentioned above regarding renewable alternative fuels’ engine combustion, performance, and exhaust emission characteristics [15]. Since the benefits offered by the modelling methodology to solve non-linear and complex issues of alternative renewable fuels in the internal combustion engine (ICE) [16]. The objective of this analysis critically reviews the application of RSM in ICE regarding the engine combustion, performance, and exhaust emission characteristics. In addition, the RSM is seen as capable of providing a relationship between the input and output variables of the experimental studies [17,18]. The basic principles of RSM are initially presented. Then, RSM applications in the ICE are reviewed [19]. Other than that, the limitations and improved solutions of RSM by combining it with other techniques are also highlighted [20,21].

### 1.1 Definition of key terms

In advance, some critical key terms are introduced and defined before conducting the review on the uses of RSM in optimizing the analytical method. Samples are also provided to clarify every single term. The experimental domain is the limit of the experiments that must be studied. The boundary of the experiment variables investigated is determined through maximum and minimum limits. The central composite, Box-Behnken, three-level factorial, and Doehlert designs are second-order experimental designs. These experimental designs define a group of variable levels that need to be implemented by the experiment to achieve the response. The experimental design refers to a particular experimental set shown via a matrix that consists of a different level combination of the variables. Independent variables, or factors, are the experimental inputs that are individually replaceable variables. Fuel blends, engine loads, and engine speed are examples of independent variables in fuel combustion experiments. variables is the different values of the variables that need to be performed for the experiments. For instance, to study the optimization of the ICE for the engine load variable, five levels of engine loads can be studied: 0 %, 25 %, 50 %, 75 %, and 100 %. The dependent, or response, variable is the experimental output variable that determines the value of the findings from the experiment. Examples of the responses are brake power, brake specific fuel consumption (BSFC) and brake thermal efficiency (BTE). Residual is the variation amongst the computed and experimental findings for a specified set of conditions. A low residual value indicates an excellent mathematical model that fits the experimental data.

## 2 Theory and methodology of RSM applications

RSM was initially established in 1951 by George E. P. Box and K. B. Wilson [22]. The RSM term had been used since the graphical viewpoint developed following the mathematical model fitness. RSM involves a group of statistical and mathematical approaches for the experimental design and model development to analyse the influence of some variables and attain the optimal condition for output responses using a small amount of experiment [23].

RSM involves the application of statistical experimental design, linear regression modelling and optimization approaches [24]. Through RSM, mathematical models produced from the experimental data describe the relationship among the output responses (dependent variables) and the input factors (independent variables). The models are utilized to evaluate the influences of input factors and the relations on the output responses and the optimization process. The findings are frequently illustrated in two-dimensional (2D) contours plots and three-dimensional (3D) plots [25].

### 2.1 Response surface methodology flow chart

**Fig. 3:**
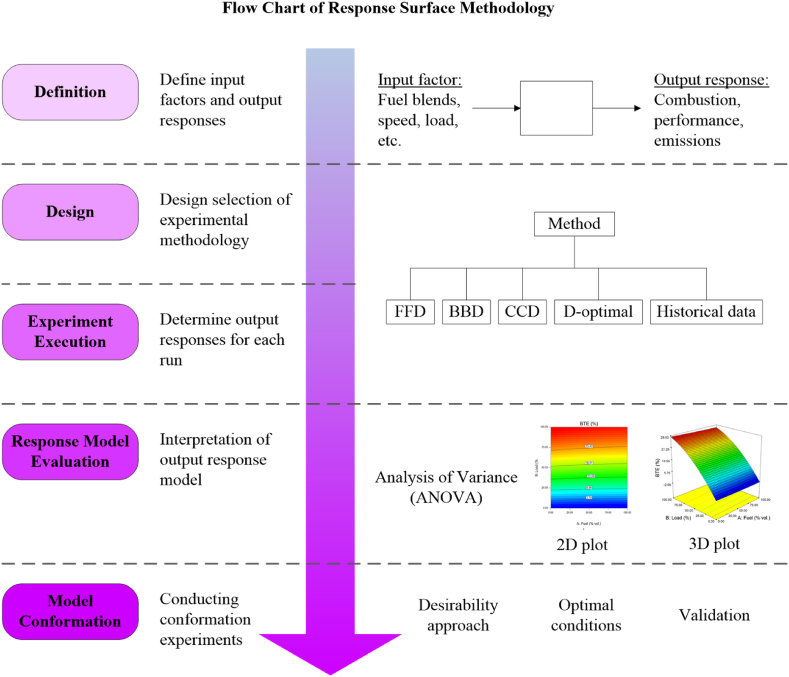
Common flow chart for RSM application.

The optimization process is a significant research field and imperative in industrialized sectors due to the product's effectiveness [26]. By utilising RSM, the research experiment and industrialized process would benefit from time-saving, low resource consumption, and less effort required. At present, there are many successful and remarkable applications of the optimization process in various areas, such as in bioprocess [27,28], analytical applications [29,30], and industrial fields [31,32]. The standard flow chart of the RSM application is as illustrated in Fig. 3.

Several stages are involved in the application of RSM as an optimization method. These consist of (i) defining of variables, (ii) design selection, (iii) experiment execution, (iv) evaluation of output response model, and (v) optimization and conformation of experiments.

### 2.2 Problem approach

The problem approach is founded on the research that uses alternative fuels in the ICE and the application of RSM. Recently, most studies related to the engine combustion, performance, and exhaust emission characteristics in the ICE have highlighted the utilization of RSM in optimizing the output findings. The researchers need to evaluate several questions on which variables will affect the results of the engine combustion, performance and exhaust emissions characteristic findings. The questions are as follow.

(i) Which type of variables must be set as constant and controlled (such as injection timing, throttle valve, engine speed, engine load, fuel blends, and so on.)?
(ii) How much gap of identified controlled variables must be carried out in every testing to be sufficient for the RSM study?
(iii) How to allocate and organize the variables so that researchers can obtain the output responses properly?
(iv) Which kind of data information in RSM would be used?

### 2.3 Definition of variables

#### 2.3.1 Selection of independent variables (factors)

A vast amount of input factors (independent variables) might influence the system's output responses (dependent variables) under study. Due to financial factors, it is nearly impractical to include, recognize and control all the minor contributions from individual variables in the experimental design. For that reason, it is essential to identify which variables that have main influences.

Screening of variables must be performed to identify which experimental variables and their interactions indicate more critical influences. Carry-out experiments commonly recognize these variables with the two-level factorial designs (full or fractional) or the one-factor-at-a-time method due to their efficiency and financial reasons [33].

The selection of appropriate levels for these input factors is also critical as it influences the model's accuracy. Commonly, two levels are used for each factor; low level (−1) and high level (+1). The difference amongst these limits is the broadest interims that can be differentiated for the study under investigation. Choosing a narrower range for these factors is essential based on the literature study and prior knowledge.

#### 2.3.2 Selection of dependent variables (responses)

In recent literature, the most common output responses of alternative fuels in ICE are the engine performances such as the exhaust gas temperature (EGT), BTE, BSFC, brake torque, brake power, and the exhaust emissions characteristics such as carbon monoxide (CO), carbon dioxide (CO_2_), hydrocarbon (HC) and oxides of nitrogen (NO_x_). It is necessary to have more than a single response that is directly connected to the aim of the research. This condition would allow the study to offer more significant facts in the analysis of the ICE. The results of the responses are expected to provide additional and further information from the optimization findings.

#### 2.3.3 Codification of variable levels

Variables codification is performed ahead of the regression analysis. Generally, codification is used to normalize the variables. It involves converting actual data into dimensionless scales, which is comparable to its investigational area. The codification is vital as it allows the analysis of different orders amount of variables with no significant effect on the assessment process. For instance, independent variables (input factors) could have different orders of units and magnitude, but all independent variables are ensured to influence the output responses equally through codification.

The transformation process of real value (z_i_) into a coded value (x_i_) is based on Equation (1) [34]:

1 $$x_{i}=\frac{z_{i}-z_{0}}{\Delta z_{i}}$$

Where.

*x*_*i*_ dimensionless coded value of independent variable,

*z*_*i*_ uncoded value of the *i*th independent variable,

*x*_*0*_ uncoded *i*th independent variable at the centre point.

*Δz*_*i*_ step change value between the low level (−1) and high level (+1).

### 2.4 Selection of design and experiment execution

Various experimental design models were employed in the ICE study, each distinct in the number of runs and experimental points. Among the frequently utilized designs are the Central Composite Design (CCD), Box-Behnken Design (BBD), full three-level factorial design, D-optimal (DO), and Historical Data Design (HDD). Typically, these statistical approaches are accessed through software like Matlab, Statistica (StatSoft), Minitab (Minitab Inc.), and Design Expert (Stat-Ease, Inc.).

In the comprehensive three-level factorial design approach, each input factor variable is set at three different levels. These levels are commonly represented as minimum, mean, and maximum, corresponding to −1, 0, and +1, respectively. This design is denoted as a 3 k factorial design, indicating the required number of experiments, 3 k, where k represents the count of independent variables. This strategy is effective for solving problems with fewer than two input factors. However, when dealing with more than two input factors, the number of necessary experimental trials for the full three-level factorial design becomes extensive, leading to the application of alternative designs.

**Fig. 4:**
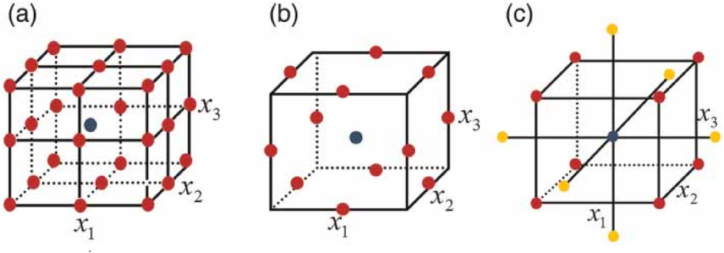
Dissimilar designs of experiment for three variables (k = 3), (a) full factorial design (three-level, n = 27), (b) Box-Behnken design (n = 15), and (c) central composite design (n = 17) where n = amount of experiment using c_p_ = 1 (a) whereas c_p_ = 3 for (b) and (c). Red point is point for factorial design, grey point indicates central points, and yellow point is axial point. Source: Nair, Makwana, Ahammed [37].

In 1960, Box and Behnken introduced the Box-Behnken Design (BBD) by combining the incomplete block design with the two-level factorial design [35]. In the BBD approach, all experimental points are situated on a sphere with a radius of √2, making it suitable for quadratic models. As depicted in Fig. 4(a–c), the BBD approach is advantageous due to requiring fewer experiments compared to the full factorial design. Since BBD does not involve experimental points at the vertices of the cubic region, it proves beneficial when investigating these points is impractical or costly for various reasons. Nonetheless, BBD comes with two constraints; the number of input factors must be three or more, and it must align with the second-order polynomial equation [36]. The number of runs in BBD is calculated using formula 2 k (k-1) + cp, where cp represents the number of repetitions at the central point and k is the count of input factors.

Box and Wilson created the central composite design (CCD) in 1951, which is analogous to the full-level factorial design technique. The CCD approach has three categories: face-centred composite, circumscribed central composite, and inscribed central composite [38]. The difference between the two designs is that CCD needs fewer experiments (runs) than the full-level factorial design. The right selection of a range of CCD techniques is determined by the experimental needs.

The CCD consists of a focal point, a two-level factorial design, and an additional design. The experiment's points are spaced apart from the experiment's centre point. 2 k plus 2 k plus the number of duplicates at the centre point determines the number of experiments. The value is often represented as = k [39]. The CCD's efficacy is quite high for up to five or six components, but thereafter rapidly declines. In addition, CCD may be employed for optimization when using a large number of parameters if the whole experiment can be conducted simultaneously (parallel) as opposed to sequentially [40].

In comparison, D-optimal (DO) is one of the RSM approaches that uses iterations to optimise algorithms. Typically, the DO technique requires a lengthy repetition for a large number of trials. Once the optimal conditions for experimental inputs and outputs have been determined [41], the iterations method is concluded. In addition, the historical data design is used to use previously measured experimental data. This approach permits the utilization of previously collected experimental data without regard to the design factor quantity [42].

#### 2.4.1 Model selection

Most RSM difficulties occur when the connection between the output responses (dependent variables) and the input factors (independent variables) is unknown. Appropriate estimation between the responses and factors is to be determined. For that reason, the initial stage in using RSM is to determine an estimation relation between the output responses and the input factors. Typically, RSM predicts a low-order polynomial. The estimation relation is the simplest first-order model if the input and output relation produces a linear function [43]. The output response and input factor can be represented as *x*_*1*_*, x*_*2*_*…, x*_*k*_, and *y*, respectively, as shown in Equation (2).

2 $$y=f^{\prime }\left(x\right)\beta +\varepsilon$$

where *x* = *(x*_*1*_*, x*_*2*_*, …, x*_*k*_*)*, *f(x)* is a vector function of *p* elements that contains powers of *x*_*1*_*, x*_*2*_*, …, x*_*k*_ to a maximum specific degree indicated by *d (>1)*. The initial *(d* = *1)* degree polynomial is written by following Equation (3).

3 $$y=\beta_{0}+\sum_{i=1}^{k}\beta_{i}x_{i}+\varepsilon$$

Where *k* is the number of factors, *β*_*0*_ is the regression coefficient for the intercept, *ε* is the residual (error) of the experiments, *x*_*i*_ represents the factors, and β_i_ is the regression coefficients of the linear parameters.

On the other hand, no curvature should be predicted by the model. If the model predicts curvature, it is necessary to use a polynomial with a higher degree, such as the second-order model. In addition, a simple linear equation (first-order model) is incapable of describing the interplay between many elements. The interaction terms of second-order quadratic equations are often implemented in RSM when the critical point cannot be identified. In addition, the two-level factorial design is used to forecast the first-order model, but it fails to account for other impacts, such as the second-order effect. Therefore, a central point in two-level factorial designs might be used to analyse curvature. The subsequent polynomial model level has additional terms that describe the relationships between the various experimental variables. Equation (4) depicts how the second-order interaction model might be stated.

4 $$y=\beta_{0}+\sum_{i=1}^{k}\beta_{i}x_{i}+\sum_{1\leq i\leq j}^{k}\beta_{ij}x_{i}x_{j}+\varepsilon$$

Where *β*_*ij*_ represents the regression coefficients of the interaction parameters.

Incorporating quadratic terms in Equation (5) is essential for the polynomial function to discover a critical point (saddle, maximum, or minimum point).

5 $$y=\beta_{0}+\sum_{i=1}^{k}\beta_{i}x_{i}+\sum_{i=1}^{k}\beta_{ii}x_{i}^{2}+\sum_{1\leq i\leq j}^{k}\beta_{ij}x_{i}x_{j}+\varepsilon$$

Where *β*_*ii*_ represents the quadratic parameter regression coefficients, this model can evaluate the interaction effect among different input factors and the critical points (minimum, maximum, or saddle point).

### 2.5 Evaluation of output response model

Once a suitable mathematical model is chosen, it is imperative to verify its predictive accuracy before employing it for forecasting. This crucial validation phase ensures the model provides a reliable approximation of the real system. The Analysis of Variance (ANOVA) serves as a dependable method for assessing the quality of the fitted model. ANOVA is crucial for contrasting the variations resulting from alterations in variable levels with those arising from the inherent random errors in the measurements of the produced outcomes. Through this comparison, assessing the significance of the regression used for forecasting responses based on the origins of experimental variance becomes possible. Various terms used during this validation process are listed in Table 1.

**Table 1:** Terminologies applied for model adequacy verification [33,38,44].

Variation source	Expression	Remarks
Coefficient of determination, R^2^	$R^{2}=\frac{SS_{\text{Re}g}}{SS_{T}}$	R^2^ must be close to 1.0
R^2^_adjusted_	$R_{adjusted}^{2}=1-\frac{SS_{\text{Re}s}/\left(n-p\right)}{SS_{T}/\left(n-1\right)}$	R^2^_adjusted_ must be close to 1.0
R^2^_predicted_	$R_{predicted}^{2}=1-\frac{PRESS}{SS_{T}}$	R^2^_predicted_ must not have a difference of more than 0.2 with R^2^_adjusted_
Prediction error sum of square (PRESS)	$PRESS=\sum_{i=1}^{n}{\left[y_{i}-{\hat{y}}_{i}\right]}^{2}$	PRESS must have a small value
Significance of regression	$F_{0}=\frac{Mean\;square\;of\;model}{Mean\;square\;of\;residual}$	This ratio must be greater than the tabulated F value for a good model
Lack of fit (LOF) test	$F_{LOF}=\frac{SS_{LOF}/\left(f-p\right)}{SS_{PE}/\left(n-p\right)}$	This ratio must be lower than the tabulated F value for a good model

The deviation within a data set is assessed by examining its dispersion via ANOVA. A common metric for describing the overall efficacy of a predictive model is the coefficient of determination (R^2^), representing the ratio of the regression sum of squares (SSReg) to the total sum of squares (SST). This ratio indicates the extent of variation in the model's predicted values from the mean. An efficient predictive model should exhibit an R^2^ value approaching 1.

However, evaluating the efficiency of model prediction should not solely rely on R^2^, as its value can increase with additional model terms, irrespective of their statistical significance. It is essential to compare R^2^ with the adjusted R^2^, which takes into account the number of experimental factors. The inclusion of statistically insignificant parameters typically results in a reduction of the adjusted R^2^ value. A substantial discrepancy between R^2^ and the adjusted R^2^ suggests the possible incorporation of non-significant terms in the model [44]. The term “residual” is crucial in assessing a model's adequacy, representing the difference between actual and predicted values. Additionally, the prediction error sum of squares (PRESS) is another vital statistic used to assess predictive capability, indicating the model's expected accuracy in forecasting future responses. A lower PRESS value is preferable. The model's predictive accuracy for new responses is represented by R^2^predicted, calculated from PRESS. A close agreement between R^2^ and R^2^predicted is desirable.

The Fisher test is employed to verify the significance of individual factors and their interactions. A higher F-value and a lower ‘p > F’ value signify greater significance of the corresponding model and its coefficients. A ‘p > F’ value below 0.05 indicates model significance at a 95 % confidence interval. The lack of fit test is another method to assess model significance, evaluating a model's inability to represent data points within the experimental domain by comparing residual error to pure error from replicated experimental design points [45]. This test should ideally be insignificant, with a proportion exceeding the charted F-value indicating a lack of fit and necessitating model refinement.

Normality assumptions can be verified through the standard probability plot of residuals. A well-fitted model will exhibit a residual plot adhering to a linear trajectory with minimal scatter, indicating normally distributed residuals. Another important assessment tool is the plot comparing experimental response points to predicted response points. Ideally, points should uniformly align along the 45° line, with clusters above or below the line indicating areas of over-prediction or under-prediction, respectively [46].

#### 2.5.1 Graphical model interpretations

Contour plots and response surface plots may be used to visualise the anticipated model equation in RSM. The contour plot is a two-dimensional (2D) representation of the fitted model, while the response surface plot is a three-dimensional (3D) graphical representation. These visualisation charts are very helpful for understanding the features of the reaction at various levels of the component. Additionally, if there are more than three input elements, the presentation of the projected model is only possible if single or extra factors are assigned a fixed value.

**Fig. 5:**
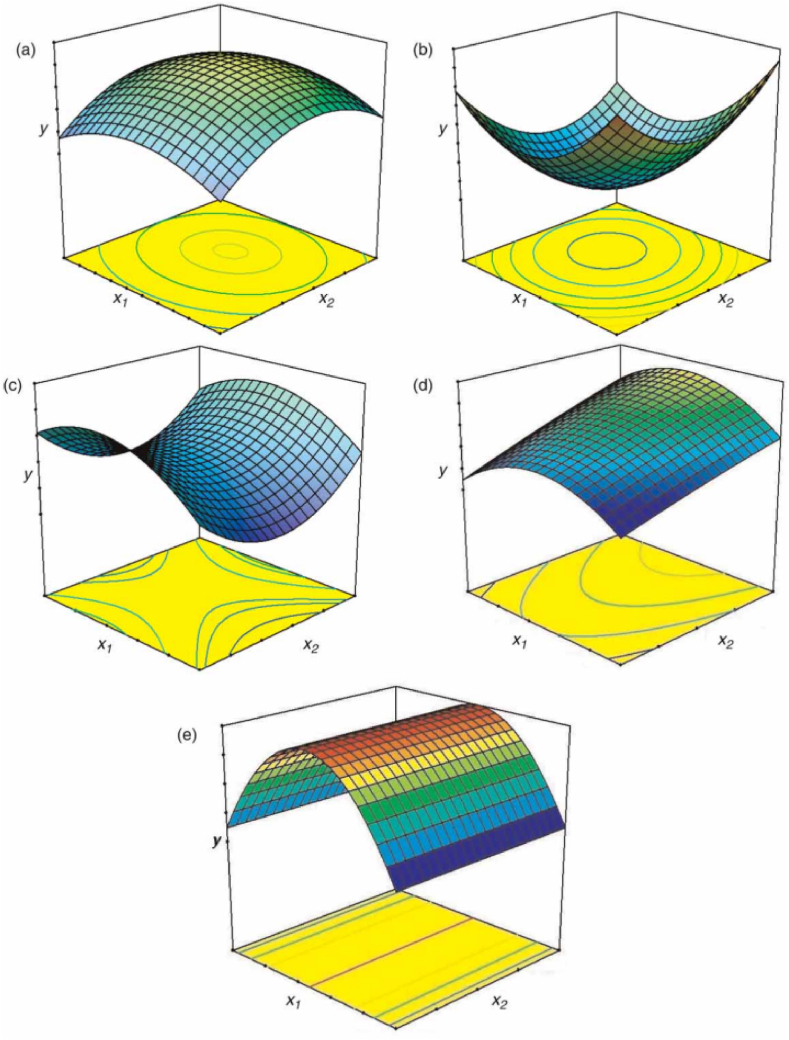
Some profiles of contour plots and corresponding response surface plots generated from a quadratic model: (a) maximum, (b) minimum, (c) saddle, (d) maximum outside the experimental region, and (e) plateau. Source: Abhilash et al. [37].

Fig. 5 shows several quadratic visualisations of anticipated model plots for optimizing two parameters in accordance with Equation (5). (a) and (b) in Fig. 5 depict 3D response surface plots. The maximum and minimum points are found inside the study region, and the circle or ellipse patterns represent the 2D contour plots. Once a hyperbolic system pattern occurs in the 2D contour plot as illustrated in Fig. 5 (c), it denotes an inflexion point amid a comparative minimum and comparative maximum. It is neither the maximum nor the lowest, and is hence known as a saddle point.

If the objective of the research is to identify a minimum or maximum reaction, the coordinates of the saddle point will not be appropriate. In addition, the optimal location of the saddle point may be determined by observing the surfaces visually. Fig. 5(d) indicates that the greatest point is situated on the perimeter of the investigated region. If at all feasible, it is necessary to adjust the preliminary model to acquire the highest possible score. The model given in Fig. 5 (e) demonstrates a plateau for variables (input factor) of x2, indicating that variations in its values have no effect on the research under consideration.

### 2.6 Optimization and conformation of experiments

Identical optimal circumstances and minimum or maximum responses may be established by analysing response surfaces. As the parameters concurrently fulfil the ideal requirements, multiple replies may be used to determine the optimal circumstances. Optimal conditions may be identified simultaneously as multiple response parameters satisfy the required criteria. In addition, the optimal condition may be determined visually by superimposing the regression model's contour plots in an overlay plot. The graphical optimization identifies potential response values in the factor space and locations that satisfy the optimization requirements [47].

In the event of more than three input elements, it might be problematic in identifying the circumstances that simultaneously fulfil the full output replies. Therefore, multi-objective optimization using the most prevalent desirability function may be used to circumvent this constraint.

#### 2.6.1 Desirability approach

The desirability method is a statistical technique employed to address real-world issues involving multiple outcomes. Its widespread use can be attributed to several advantages, including the ease of assigning significance and weights to individual outcomes, accessibility through various software platforms, user-friendliness, and adaptability for modifications. This method consolidates multiple outcomes into a singular, dimensionless performance metric known as the desirability function [48].

This technique entails converting each predicted outcome Y_i_, which fluctuates within the range 0 < d_i_ < 1, where the value of di denotes the desirability of the corresponding response Y_i_. For example, a di value of 1 signifies a completely acceptable response, whereas a di value of 0 denotes a wholly undesirable one [49]. The objective for each response can vary – it may be to minimize, maximize, target, equate to, or fall within a specific range, contingent on the problem's nature. The desirability of individual responses, in relation to the objective of each, can be calculated using specific equations. For a goal of minimum response, the desirability d_i_ will be defined as in Equation (6) to Equation (14) [50],

6 $$d_{i}=1,\text{when}Y_{i}\leq Low_{i}$$

7 $$d_{i}={\left(\frac{High_{i}-Y_{i}}{High_{i}-Low_{i}}\right)}^{wt_{i}},\text{when}Low_{i}<Y_{i}<High_{i}$$

8 $$d_{i}=0,\text{when}Y_{i}\geq High_{i}$$

For a goal of maximum response, the desirability d_i_ will be defined as,

9 $$d_{i}=0,\text{when}Y_{i}\leq Low_{i}$$

10 $$d_{i}={\left(\frac{Y_{i}-Low_{i}}{High_{i}-Low_{i}}\right)}^{wt_{i}},\text{when}Low_{i}<Y_{i}<High_{i}$$

11 $$d_{i}=1,\text{when}Y_{i}\geq High_{i}$$

For a goal as target response, the desirability d_i_ will be defined as,

12 $$d_{i}=0,\text{when}Y_{i}<Low_{i},Y_{i}>High_{i}$$

13 $$d_{i}={\left(\frac{Y_{i}-Low_{i}}{T_{i}-Low_{i}}\right)}^{wt_{i}},\text{when}Low_{i}<Y_{i}<T_{i}$$

14 $$d_{i}={\left(\frac{Y_{i}-High_{i}}{T_{i}-High_{i}}\right)}^{wt_{i}},\text{when}T_{i}<Y_{i}<High_{i}$$

For the goal within the range, *d*_*i*_ = *1* when *Low*_*i*_ *< Y*_*i*_ *< High*_*i*_; and *d*_*i*_ = *0*; otherwise.

Here *i* specifies the response, *Y* the value of the response, *Low* indicates the lower limit of the response, *High* indicates the upper limit of the response, *T* represents the target value of the response, and *wt* means the weight of the response. The weight field can alter the desirability function shape for each response. Weights are used to highlight the lower/upper bounds specifically. Weights can range from 0.1 to 10; a weight higher than 1 emphasises the objective and weighs less than one gives lower. When the weight value is equal to 1, the desirability function varies in a linear model. Solving of multiple response optimizations using the desirability approach involves a method of combining various responses into a dimensionless performance measurement called the overall desirability function, D (0 ≤ D ≤ 1), based on Equation (15). 15

15 $$D={\left(\prod_{i=1}^{n}d_{i}^{r_{i}}\right)}^{1/\sum r_{i}}$$

Within the comprehensive desirability objective function (D), each response can be allocated a level of importance (r) in comparison to others. The significance assigned ranges from a minimum value of 1, represented by (+), to a maximum critical value of 5, denoted by (+++++). A higher D value signifies a more desirable system with optimal functions, representing the best solution. The optimal values of the factors are ascertained by the individual functions (d) value that maximizes D. Solutions derived through the desirability method are subsequently validated through confirmatory experimental tests, which are conducted based on established optimization criteria.

#### 2.6.2 Conformation experimental

The confirmation experiment is required to check the model's accuracy between the observed experimental data and the projected value of the regression analysis. According to Kumar et al. [51], confirmation trials are unnecessary if the RSM models yield fewer than 5 % prediction errors. Furthermore, using Equation (16), the percentage of absolute error (POAE) is utilized to assess the discrepancy between the observed experimental findings and the projected value.

16 $$\text{Percentage}\;\text{of}\;\text{Absolute}\;\text{Error}\left(\%\right)=\left(\frac{\text{Actual}\;\text{value}\;-\;\text{Predicted}\;\text{value}}{\text{Actual}\;\text{value}}\right)\times 100$$

## 3 Application of RSM in internal combustion engines

In recent years, the majority of studies in ICE have emphasised the significance of RSM as an extra analytic tool for maximising the number of data collected from the same amount of experimental data. Numerous researchers have successfully used this strategy to enhance engine combustion, performance, and exhaust emission by using a wide variety of engine settings and alternate fuels. The bulk of the researchers and practitioners in this domain concentrated on utilising RSM to answer the single and multiple input elements with the output replies. Box and Draper [52] theorised that optimization using RSM was first associated with the output responses of the experimental model and later evolved into the modelling of numerical experiments. Satake et al. [53] revealed that by using the RSM, about 54% of the person-hour trial could be decreased.

Recent review articles discuss the application of RSM in several fields, including analytical chemistry [54], chemical and biochemical process [55], electroanalytical chemistry [56], analytical scale HPLC separation [57], renewable and sustainable energy [58], Fenton oxidation process [59], chromatographic systems [60], genetic algorithms [61], structural optimization [62], water and wastewater treatment processes [37,63], biometric [64], and energy [65]. Although there have been various analyses of RSM, research on engine applications is insufficient. Therefore, a comprehensive review of the use of RSM in ICE must focus only on engine combustion, performance, and exhaust emissions. Table 2 contains a summary of significant RSM-based findings made by the ICE.

**Table 2:** **V**arious RSM applications in the research of the ICE.

Group	Fuel Type	Engine Type	Input Factor (Independent Variable)	Output Response (Dependent Variable)	X^Y^ Design	Run	Optimization	Overall Desirability	POAE (%)	Ref.
Engine Parameters	DF	CI, 4 S, 1-cyl, DI, AC	ES, EL, IT	BSFC, HC, NO_x_, smoke, noise	3^4,4,3^ FFD	48	ES: 2076 rpm, EL: 5.6 N m, IT: 26 °CA bTDC	–	–	[66]
	DF	CI, DI	CR, IT, IP, EGT	Pmax, NO_x_, smoke	4^3^ BBD	27	CR: 14.25, IP: 1153.15 bar, IT: 13.69 °CA bTDC, EGR: 16.91 %.	0.95	<10	[67]
	DF	CI, 1-cyl, DI	IP, BP, IT, EGR	BSFC, NO_x_, PM	4 F_r_FD	12	IP: 90 MPa, BP: 155.1 kPa, IT: 1 °CA aTDC, EGR: 21.4 %	–	–	[68]
Alcohols	DF-Water hyacinth plant (*Eichhornia crassipes*) bio-Eth	CI, 4 S, 1-cyl, WC, VCR	EL, CR, IP	Pmax	3^3^ CCD	20	FB: 5 %, 10 %	–	<2	[69]
	DF-Hydrous Eth	RCCI, 4 S, 1-cyl, DI	SOIC2, Inj1Fr, FEF, P_rail_, P_int_, Dwell, T_int_, EGR	CO, CO_2_, HC, NO_x_, smoke	F_r_FD	<60	–	–	–	[70]
	GF-Bio-Eth (potato peel wastes)	SI, 4 S, 4-cyl	FB, ES	Torque, Bp, BSFC, CO, CO_2_, HC, NO_x_	2^5^ CCRD	45	FB: 10 %, ES: 3000 rpm	0.98	<4	[71]
	GF-Eth	SI, 4 S, 1-cyl, AC, NA	FB, EL, ES	FCE, CO, NO_x_	3^3^ BBD	17	Eth: 3.92–4.12 %, EL: 30 %, ES: 57 km/h	0.72	<11	[72]
	GF-Iso-Bu	CI, 4 S, 1-cyl, DI, NA	IP, IT, EGR	BSFC, BTE, CO_2_, NO_x_, smoke	3^3^ FFD	27	IP: 240 bar, IT: 23 °CA bTDC, EGR: 30 %	0.969	4	[73]
	GF-2-Bu	SI, 4 S, 4-cyl, IDI, NA	FB, ES	Bp, BMEP, BSFC, BTE, CO, CO_2_, HC, NO_x_	–	13	FB: Bu15, ES: 3205 rpm	0.8	<10	[74]
	GF-Fusel oil	SI, 1-cyl, WC, IDI	FB, EL	Torque, BSFC, BTE, CO, HC, NO_x_	2^5^ -	13	FB: 25 %, EL: 47.21 %	0.63	<4	[75]
	GF-Fusel oil	SI, 4 S, 4-cyl, IDI, NA	FB, EL, ES	Bp, BSFC, BTE, CO, HC, NO_x_	3^4^ UDD	64	FAWE: 20 %, EL: 55.4 %, ES: 4499 rpm	0.707	<5	[76]
	GF-*n*-Oct	CI, 4 S, 1-cyl, DI, WC	FB, EGR, IT	Smoke, NO_x_, BTE, BSFC,	3^3^ FFD	27	FB: 17 %, EGR: 10 %, IT: 20 °CA bTDC	0.967	<4	[77]
	DF- Bu, Pe, Prop	CI, 4 S, 1-cyl, DI, NA	FB, IT, EGR	Smoke, NO_x_, HC, CO, BTE, BSFC,	3^3^ FFD	27	FB: n-propanol, IT: 25 °CA bTDC, EGR: 30 %	0.965	<14	[78]
Biofuel	DF-Hermetia illucens larvae oil	CI, 4 S, 1-cyl, DI	FB, EL	Bp, BMEP, BSFC, CO, CO_2_, HC, NO_x_	2^5^ -	25	FB: 6.43 %, EL: 92.72 %	0.819	<7.5	[79]
	DF-Waste biomass pyrolysis oil	CI, 1-cyl, DI, WC	FB, CR, EL	BSFC, BTE, CO, CO_2_, HC, NO_x_, smoke	3^3^ FFD	27	FB: 20 %, CR: 18, EL: 100 %	0.7018	5	[80]
	DF-Waste plastic pyrolysis oil	CI, 4 S, 6-cyl, DI, WC, NA	FB, EL, ES	Torque	2^2,4,5^ FFD	60	–	–	10	[81]
Biodiesel	DF-Bd	CI, 4-cyl, DI	FB, EL, ES	HC, CO, NO_x_	3^5^ CCRD	–	FB: 77.8 %, EL: 41.25 %, ES: 2800 rpm	–	–	[82]
	DF-Argemone Mexicana (FAME)	CI, 4 S, 1-cyl, VCR	FB, EL, CR	BSFC, BTE, CO, HC, NO_x_	3^4^ FFD	64	FB: 20 %, EL:9.8 kg, CR:18	0.97	8	[83]
	DF-Calophyllum inophyllum (FAME)	CI, 4 S, 1-cyl, VCR, WC	EL, CR	BSFC, η_mech_, BTE	2^4,5^ UDD	20	EL: 6 kg, CR: 18	–	<7	[84]
	Canola oil (FAME)	CI, 4 S, 4-cyl, DI, TC	ES, IT	Torque, Bp, BMEP, BSFC, BTE, EGT, O_2_, NO_x_, CO_2_, CO, LAC	2^3^ CCFCD	9	–	–	–	[85]
	DF-Cassia tora (FAME)	CI, 4 S, 1-cyl, DI	FB, EL, IT, IP	BTE, HC, NO_x_	4^5^ CCRD	31	FB: 40 %, EL: 47 %, IT: 15 °CA bTDC, IP: 221 bar	0.967	5	[86]
	DF-Hazelnut oil (FAME)	CI, 4 S, 4-cyl, DI, TC	IT, ES	Bp, BSFC, BTE, EGT, NO_x_, CO_2_, CO, smoke	2^3^ CCFCD	9	–	–	–	[87]
	Honge oil (Bd)	CI, 4 S, 1-cyl, CRDI, WC	EGR, IP, IT	P_max_, HRR, ID, CD, BTE, CO, HC, NO_x_, smoke,	3^3^ FFD	27	EGR: 21 %, IP: 900 bar, IT: 10° CA bTDC	–	–	[88]
	DF-Honge (FAME)	CI, 4 S,1-cyl, WC	FB, EL, IT, CR	BTE, NO_x_	4^3^ CCD	31	EL: 86.3 %, FB: 15 %, CR: 16, IT: 26.24 °CA bTDC	0.6342	<4	[89]
	Jatropha (Bd)	CI, 1-cyl, DI, WC	10 parameters	BTE	10^2^ TD	16	–	–	–	[90]
	DF-Jatropha curcas shell bio-oil (pyrolysis)	CI, 4 S, 1-cyl, WC, VCR	FB, CR, EL	BSFC, BTE, CO, CO_2_, HC	3^3^ CCD	20	FB: 12.22 %, CR: 18, EL: 6.665 kg	0.786	<27	[91]
	DF-Jatropha curcas (Bd)	CI, 4 S, 4-cyl, DI	EL, IP, IT, II	BSFC, BTE, CO, HC, NO_x_, soot	4^3^ FFD	81	EL: 65.71 %, IP: 160 MPa, IT: 4.02 °CA bTDC, II: 4.40 °CA	0.866	<6	[92]
	DF-Jatropha curcas (Bd)	CI, 4-cyl, WC, TC	IP, IT, II	NO_x_, HC, BSFC, BTE, soot	3^3^ F_r_FD	80	IP: 134.11 MPa, IT: 6.4 °CA bTDC, II: 5.8 °CA	0.967	<4	[93]
	DF-Jatropha (Bd)	CI, 4 S, 1-cyl, DI, WC, VCR	FB, EL, CR	Pmax, BSFC, BTE, CO, HC, NO_x_, smoke	3^5^ CCRD	20	FB: 17.35–22.63 %, EL: 7.98–11.62 N m, CR: 14.92–15.80	–	<5	[49]
	DF-Mahua oil (FAEE)	CI, 4 S, 1-cyl, DI, WC, VCR	CR, FB, EL, IT, ES	BSFC, BTE, Pmax, CO, HC, NO_x_, smoke	5^5^ CCRD	32	FB: 23 %, EL: 75 %, IT: 345 CAD, ES: 2920 rpm, CR: 15.50	0.7364	<2	[94]
	DF-Pongamia (FAME)	CI, 2-cyl, DI, WC, NA	IP, IT, NTP	BSEC, BTE, CO, HC, NO_x_, smoke	3^5,5,3^ F_r_FD	50	IP: 225 bar, IT: 21 °CA BTDC, NTP: 2.5 mm	0.98	<3	[48]
	DF-Pongamia (FAME)	CI, 4 S, 1-cyl, DI	FB, IP, IT, EL	BTE, HC, NO_x_	4^5^ CCRD	31	FB: 40 %, IP: 196.36 bar, IT: 15 °CA bTDC, EL: 53 %	0.967	<5	[95]
	DF-WCO (FAEE)	CI, 4 S, 1-cyl, DI, NA, AC	FB, EL	η_Energy_, η_Exergy_, EGT	2^11,10^ -	–	EL: 25–30 %	–	–	[96]
	DF-WCO (FAME)	CI, 4 S, 4-cyl, DI, TC, WC, NA	FB, EL, ES	Torque, Bp, BSFC	3^5^ CCRD	20	–	–	–	[97]
	DF-WCO (FAME)	CI, 4 S, 1-cyl, DI	CR, IP, IT	BTE, BSFC, EGT, smoke	3^4,3,3^ HDD	36	CR: 17.99, IP: 250 bar, IT: 27°CA bTDC	0.778	–	[98]
Additive	DF-DEE, DMC, DGM	CI, 1-cyl, DI, AC, NA	FT, FB, IT	BSFC, BTE, CO, HC, NO_x_, smoke	3^3^ TD	9	FT: DGM, FB: 10 %, IT: 21 °CA bTDC	–	–	[99]
	DF-DMC, iso-Bu, *n*-Pe	CI, 4 S, 1-cyl, DI, NA	FB, EGR, IT	BSFC, CO, HC, NO_x_, smoke	3^3^ FFD	27	FB: DF-iso-Bu, IT: 22 °CA bTDC, EGR: 0 %	0.988	5	[51]
Gaseous	DF-H_2_	CI, 4 S, 4-cyl, DI, WC, TC, IC	FR, EL	BTE, CO, HC, NO_x_	2^6,4^ GFD	24	–	–	–	[100]
	DF-Producer gas (rice husk)	CI, 4 S, 4-cyl, DI, WC, TC, IC	FR, EL	BTE, CO, HC, NO_x_	2^6,4^ GFD	24	–	–	–	[101]
	CNG - Honge oil (FAME)	CI, 4 S, 1-cyl, DI, WC	CR, FR, IT	P_max_, HRR, ID, CD, BTE, CO, HC, NO_x_, smoke	3^3^ FFD	27	–	–	–	[102]
Ternary Fuels	DF-GF-WCO (FAME)	HCCI, 1-cyl, DI, NA	FB, EL, FR	BTE, CO, HC, NO_x_, smoke	3^3^ CCD	30	FB: Bd100, EL: 48.0524 %, FR: 0	0.7771	<19	[103]
	DF- Eth-WCO (Bd)	CI, 4-cyl, WC	FB, EL, ES	CO, CO_2_, HC, NO_x_, smoke	4^5^ CCRD	31	DF: 63 %, Bd: 26 %, Eth: 11 %, EL: 80 %, ES: 2800 rpm	0.74	–	[104]
	DF-Eth -Soybean oil (FAME)	CI, 4 S, 4-cyl, DI, WC	FB, EL, ES	BEE	4^5^ CCRD	31	FB: Bd17Et8, ES: 1900 rpm, EL: 94 %	–	–	[105]
	DF-Bu-cotton oil	CI, 4 S, 4-cyl, DI, TC	FB	Torque, Bp, BSFC, BTE, BMEP, CO, HC, NO_x_	3^7^ -	–	DF: 65.5 %, Bu: 23.1 %, B: 11.4 %	0.83513	<1	[106]
	DF-*n*-Bu-Cotton oil	CI, 4 S, 4-cyl, DI, TC	FB	Torque, Bp, BTE, BMEP, BSFC, NOx, CO, HC	3^7^ HDD	7	DF: 65.5 %, *n*-Bu: 23.1 %, B: 11.4 %	–	<9	[107]
	DF-WCO, *n*-Bu, *n*-Pe, n-Prop	CI, 4 S, 1-cyl, DI, WC	FB, IT, EGR	BSFC, BTE, CO, HC, NO_x_, smoke	3^3^ FFD	27	FB: DF50-WCO30-Pe20, IT: 23 °CA bTDC, EGR: 15 %	0.974	6	[108]
	DF-Pe-Calophyllum inophyllum (Bd)	CI, 4 S, 1-cyl, DI, WC, VCR	FB, EL, CR	BSFC, BTE, CO, CO2, HC, NO_x_, smoke	3^3^ FFD	27	Pe: 20, Bd: 20, EL: 2.5 BMEP, CR: 17.5	0.88	<10	[109]
	DF-DEE-Aegle marmelos (bael) neat oil	CI, 4 S, 1-cyl, DI, NA, WC, VCR	FB, CR, IP, IT	BSFC, BTE, CO, HC, NO_x_, smoke	4^3^ FD	81	–	0.6168	5	[110,111]
	DF-DEE- Chaulmoogra neat oil	CI, 4 S, 1-cyl, DI, NA, WC, VCR, EGR	CR, ES, EGR	BSFC, BTE, CO, HC, NO_x_, smoke	3^4^ FD	64	EGR: 10 %, CR: 18.1, ES: 1672 rpm	0.665	<5	[112]
	DF-DEE-Chaulmoogra neat oil	CI, 4 S, 1-cyl, DI, NA, WC, VCR, EGR	CR, ES, EL, EGR	BSFC, BTE, CO, CO_2_, HC, NO_x_, smoke,	4^4^ -	–	–	0.628	5	[113]
	DF-DEE-Bd	CI, 4 S, 1-cyl, DI, WC	CR, IP, IT	BTE	BBD	15	CR:18, IP: 220 bar, IT: 20 °CA bTDC	–	<1	[114]
	DF-Calophyllum inophyllum (FAME)-BHA, BHT, TBHQ, PG, PY	CI, 4 S, 1-cyl, DI, WC, NA, VCR	IT, CR	BSFC, BTE, CO, CO_2_, HC, NO_x_, smoke	2^4^ FFD	16	FB: B20D3 CR: 17.5, IT: 23 °CA bTDC	0.785	5	[50]
	DF-Bd-H_2_	CI, 4 S, 1-cyl, WC	FB, FR, EGR	BSFC, BTE, NO_x_, smoke	3^3^ TD	27	FB: 20 %, FR: 30 %, EGR: 40 %	–	–	[115]
	DF-Eth-Hex-DEE	CI, 4 S, 1-cyl, IDI, AC	FB, EL	BSFC, CO, HC, NO_x_	2^-^ DO	19	Eth: 40 %, Hex: 5 %, DEE: 15 %, EL: 95 %	–	<18	[116]

### 3.1 Engine parameters

An investigation was performed by Win et al. [66] using a 3.5 kW compression ignition (CI) engine as the experimental engine powered by diesel fuel (DF). The RSM model was evaluated to explore the influences of its static injection timing (IT) at (26 °CA, 32 °CA, and 38 °CA) bTDC, engine speeds (ES) varied at (1500, 2000, 2500, and 3000 rpm), and engine load (EL) torque at (40 %, 60 %, 80 %, and 100 %) of rated value. The output responses are radiated engine noise, smoke, NO_x_, HC, and BSFC. They ran 48 experiments for a complete set of investigations based on three factors with 4 × 4 × 3 levels of full factorial design (FFD) array analyzed in this research. It had been discovered that the optimum point for the regression model was at 2076 rpm of engine speed, 5.6 N m of engine loads, and 26 °CA bTDC with the corresponding responses of 346 g/kW h for BSFC, 47 ppm for HC, 346 ppm for NO_x_, 2 HSU for smoke, and 90.1 dB for engine noise.

Ganji et al. [67] investigated the effect of engine parameters using a Caterpillar 3401 direct injection (DI) diesel engine. The Box-Behnken design is based on input factors of different compression ratio (CR) (12, 14.25, and 16.5), the start of injection (SOI) (0° CA, 15° CA, and 30° CA) bTDC, fuel injection pressure (500 bar, 950 bar, and 1400 bar), and exhaust gas circulation (0 %, 12.5 %, and 25 %). The outcome from that study revealed that at a condition of 14.25 compression ratio, 1153.15 bar fuel injection pressure, SOI 13.69 ° CA bTDC start of injection pressure, and 16.91 % exhaust gas recirculation (EGR) is the optimum combination of the operating parameter. The RSM discovered the composite desirability of 0.95.

Lee and Reitz [68] have investigated the emission reduction ability of EGR and other parameters on a single-cylinder, high-speed direct-injection (HSDI) diesel engine equipped with a common rail injection system. They used fractional factorial design (FrFD) of the RSM method in the study. It is the most effective method to reduce the number of experiments while still obtaining the desired data information. The injection pressure (IP), boost pressure (BP), injection timing (IT), EGR rate were selected as the input factor. In contrast, particulate matter (PM), NO_x_ and BSFC were selected for the output responses to obtain modulated kinetics combustion region (low-temperature and premixed combustion regime). It is significant to note that the best optimum parameters were obtained at 90 MPa injection pressure, 155.1 kPa boost pressure, 1.0 °CA after top dead centre (aTDC) of SOI, and 21.4 % EGR rate.

### 3.2 Alcohols

Choudhary et al. [69] used a four-stroke, single-cylinder diesel engine to evaluate the combustion characteristics and performances of these test fuels during a research of bioethanol derived from the water hyacinth plant (*Eichhornia crassipes*) in blended diesel fuels. The RSM method was used to examine the effects of fuel injection pressures (180 bar, 225 bar, and 270 bar), engine loads (6 kgf, 8 kgf, and 12kgf), and compression ratios (14, 16, 18) on the output response of peak in-cylinder pressure at various diesel-bioethanol blends (5%, 10%, 15%, 20%, and 25%) (Pmax). The CCD data indicated that the most significant diesel-bioethanol blended ratio was between 5 and 10% bioethanol to diesel, resulting in a large combustion peak pressure in comparison to other blends.

Fang et al. [70] conducted an array of trials. In a modified single-cylinder Isuzu 4HK1-TC DI, reactivity-controlled compression ignition (RCCI) engine, hydrous ethanol was mixed with diesel fuel. This investigation's methodological strategy was developed in accordance with the FrFD of RSM. In this study, the input component was divided into two sets of experiments: low engine load (4.5 bar gross of indicated mean adequate pressure, IMEP) and high engine load (10 bar gross of IMEP). At low engine load condition, the input factor was indicated as the start of injection command for the 2nd injection (SOIC2), injection fraction of first diesel injection (Inj1Fr), diesel fuel rail injection (Prail), intake air pressure (Pint), and fumigant energy fraction (FEF). While at high engine load conditions, the input factor is identified as the time interval between two diesel fuel injections (Dwell), Inj1Fr, Prail, intake air temperature (Tint), and EGR. This FrFD study is comprised of less than sixty experimental point tests at each engine load level. This study gives a thorough comprehension of the optimization of complicated dual-fuel RCCI combustion modes in CO, CO2, HC, NOx, and soot. The researchers identified a possibility to dramatically reduce bioethanol's life-cycle energy usage and CO2 emissions.

Using RSM analysis, Najafi et al. [71] examined the utilization of bioethanol generated from potato peel wastes combined with gasoline fuel (GF) at various blending ratios (5 %, 7.5 %, 10 %, 12.5 %, and 15 %) in a four-cylinder, four-stroke, KIA 1.3 SOHC gasoline engine. Fuel blends (FB) at varying engine speeds (2000 rpm–4000 rpm with a 500 rpm interval) are the input factor for this investigation, which includes 45 separate tests. NOx, HC, CO2, CO, BSFC, torque, and power are chosen as the output responses fulfilled by the rotatable central composite design (CCRD). A blend of 90 % gasoline fuel with 10 % bio-ethanol at 3000 rpm engine speed was found to be at the optimal input parameters with maximum desirability of 0.98.

Chen et al. [72] completed a rigorous investigation of exhaust pollution and engine performance optimization using the Box-Behnken design. In that investigation, a 125 cc four-stroke, single-cylinder, air-cooled motorcycle engine with an original carburettor and driven by gasoline with ethanol-blended fuels was employed. As input factors, three kinds of variables were chosen: ethanol blends ratios, throttle positions, and motorcycle speed, which were chosen by 0 %, 10 %, and 20 % by volume of ethanol-gasoline blends, 30 %, 60 %, and 90 % of throttle position, and 30, 45, and 60 % of speed, respectively. The experimental trials' output responses were fuel conversion efficiency (FCE) and CO and NOx emissions. This study will look at the best conditions for the highest FCE and if minimal air pollution emissions can be obtained using RSM. The ideal point was discovered utilising a 3.92% to 4.12% ethanol mix in gasoline fuel, at 30 % throttle position and 57 km/h speed, with the greatest desire value of 0.72. Because of the lower heating value of ethanol, the FCE value was enhanced by 23.6% at optimal circumstances, according to the output answers. In contrast, CO and NOx emissions were decreased by 29 % and 12 %, respectively, when compared to gasoline fuel.

Saravanan et al. [73] used a three-factor-three-level (33) complete factorial design to optimise 40% by volume of iso-butanol (Bu) and diesel mix fuel to analyse engine performance and exhaust emissions at 1500 rpm CI engine. EGR, injection pressure, and injection time were employed as input variables in that researcher's study to evaluate utilising RSM that were plotted using constructed regression models. Finally, the research found that an iso-butanol/diesel fuel mix with 30% EGR, injection pressure of 240 bars, and injection timing of 23 °CA bTDC were optimal. It provided the greatest BTE while minimising BSFC, NOx, and smoke emissions, with a desirability of 0.969 and a forecast error of 4 %.

Yusri et al. [74] used RSM to investigate the influence of gasoline mix fuels including 2-butanol (at 5%, 10%, and 15% by volume). The gasoline test engine was run at half throttle (WOT) and at varied engine speeds (2000 rpm–4000 rpm with intervals of 500 rpm). The NOx, HC, CO2, CO, BTE, BSFC, BMEP, and braking power output responses were investigated utilising a Mitsubishi 4G93 single overhead camshaft (SOCH), four-stroke, four-cylinder, indirect injection (IDI), spark ignition (SI) engine. The researchers discovered that 85% volume of gasoline fuel combined with 15% volume of 2-butanol at an engine speed of 3205 rpm was the best option using a maximum desirability based technique of 0.8. Furthermore, improvements in braking power, BMEP, BTE, CO, HC, and NOx were discovered. However, BSFC showed a minor increasing tendency, although CO2 emissions were greatly increased.

Ardebili et al. [75] employed a new RSM approach to optimise engine performance and emissions utilising alcohol-based biofuel derived from fusel oil in a single-cylinder, four-stroke, port fuel injection (PFI) gasoline engine (Ricardo Hydra). The key input elements were varied gasoline fuel and fusel oil mixes (0%, 25 %, 50 %, 75 %, and 100 %) under varying engine loads (20 %, 40 %, 60 %, 80 %, and 100 %) and at a constant engine speed of 2500 rpm. NOx, HC, CO, BTE, BSFC, and torque were all measured as output responses. A total of 13 experiments were conducted concerning the group of input elements and output reactions. An ideal solution with a high desirability of 0.63 was identified employing 25 % fusel oil in gasoline fuel at a 47.21% engine load.

Awad et al. [76] used a Mitsubishi 4G93 SOCH 4-stroke 4-cylinder naturally aspirated port fuel injection SI engine with gasoline fuel-fusel oil mixtures in their user-defined design (UDD) analytical investigation. The gasoline fuel-fusel oil blends were studied at 10 % and 20 % blending percentages, respectively. In contrast to the other study, this investigation concentrated on the numerous input elements, especially varied engine speeds (1500, 2500, 3500, and 4500 rpm) and various throttle valve opening positions at 15 %, 30 %, 45%, and 60 %. The optimal point of the input variables was discovered utilising fusel oil after water extraction (FAWE) fuel blends at 20 %, engine speed of 4499 rpm, and throttle position of 55.4%. Furthermore, a maximum desirability of 0.707 was recorded, with a validation percentage of absolute error of less than 5 %.

Gopal et al. [77] investigated the prediction of engine performance and exhaust emissions in a single cylinder, four strokes, DI diesel engine utilising diesel fuel and *n*-octanol mixtures. The researchers presented the implementation of multi-objective optimization utilising RSM based on 3 × 3 complete factorial design matrices with varied fuel mix ratios (10 %, 20 %, and 30 % *n*-octanol), injection timing (19°, 21°, and 23° CA bTDC), and EGR (10 %, 15 %, and 20 %) as input parameters. RSM was used to create a model for BFSC, BTE, NOx, and smoke, and the findings were statistically significant. The ideal condition was discovered utilising a 17% diesel fuel/n-octanol fuel mix, injection timing of 20° CA bTDC, and EGR of 10 %, with a desirability of 0.967 and acceptable precision within 4 %.

Kumar et al. [78] investigated three high carbon bio-alcohol/diesel fuel mixes. They made it by combining 40 % by volume of *n*-pentanol (Pe), n-butanol, and n-propanol with diesel fuel in a naturally aspirated, single-cylinder, four-stroke, CI engine. The RSM was built using a complete factorial design matrix with 33 (three-factor, three-level) factors. It employed alcohol type, injection timing (21°, 23°, and 25° CA bTDC), and EGR rate (10 %, 20 %, and 30 %) as input elements to test six output responses of BSFC, BTE, CO, HC, NOx, and smoke.

### 3.3 Biofuel and biodiesel

Sakthivel et al. [80] concentrated their investigation on the impact of waste biomass pyrolysis oil and diesel fuel. The authors conducted experiments on the Kirloskar single-cylinder DI engine. The bio-oil ratio was blended with several diesel fuel blends (10 %, 15 %, and 20 % bio-oil), varied engine loads (50 %, 75 %, and 100 %), and varying compression ratios (16:1, 17.5:1, 18:1). It was intended to be the input factor for research into their relationship with BSFC, BTE, CO, CO2, HC, NOx, and smoke opacity responses. RSM's complete factorial design revealed that the optimal condition parameters were 20% bio-oil concentration, 100% engine load, and 18:1 compression ratio with an error of prediction of less than 5 % and 0.7018 composite desirabilities.

Poompipatpong and Kengpol [81] provided a decision-making process based on the average value and the RSM. The engine torques might be compared using this manner. As input elements, the inquiry employed several fuel mixes of diesel fuel with waste plastic pyrolysis oil (0% and 25 %), engine speed (800 rpm, 1000 rpm, 1200 rpm, 1500 rpm, 1800 rpm, and 2000 rpm), and engine load (20 %, 40 %, 60 %, 80 %, and 100 %). Based on the complete factorial design of the experiment, a four-stroke, six-cylinder, DI heavy-duty Hino W06D diesel engine was employed in this study. The researchers reported on a more accurate way for determining engine torque that they had found. Furthermore, the number of tests may be minimised, which saves time and money for future study.

Alireza Shirneshan et al. [82] developed regression models for CO, HC, and NOx tail emission on four-cylinder, DI diesel engines in terms of diesel fuel-biodiesel mixes, engine load, and engine speed. According to the study, the best condition was discovered to be 77.8% biodiesel in diesel fuel, 41.25% engine load, and 2800 rpm engine speed based on the three-factor five-level central composite rotatable design.

Parida et al. [83] studied the effect of Argemone Mexicana fatty acid methyl ester (FAME) in a single-cylinder, four-stroke, multi-fuel variable compression ratio (VCR) engine. The experiment was designed to optimise parameters such as BSFC, BTE, CO, HC, and NOx. The operating parameters of fuel mixes (20 %, 40 %, 60 %, and 100 %), compression ratios (16, 17, 17.5, and 18), and engine loads (3 kg, 6 kg, 9 kg, and 12 kg) were chosen as the key elements influencing the optimal responses. The three-factor four-level RSM design with 64 total runs was employed in the research based on the analytical investigations via the complete factorial design matrix. The researchers found a composite desirability of 0.97009 for the optimal outcome at 20 % fuel mix ratio, 18.0 compression ratio, and 9.8 kg engine load.

Venugopal et al. [84] investigated the impact of various compression ratios (14, 15, 16, 17, and 18) and engine load conditions (1 kg, 2 kg, 4 kg, and 6 kg) utilising 25 % *Calophyllum inophyllum* (hone oil) methyl ester and 75 % diesel fuel mix. A four-stroke VCR CI engine was used for the experiment. Based on the user-defined RSM design, the output answers were particular fuel consumption, BTE, and mechanical efficiency (mech). According to the approach, the engine load of 6 kg with an 18:1 compression ratio was the ideal parameter condition for obtaining superior engine performance characteristics.

Based on their investigation, different researchers chose distinct types of input elements. The chosen input parameters in the Ileri et al. [85] investigation were engine speed (2000, 3000, and 4000 rpm) and fuel injection timing (12°, 15°, and 18° CA bTDC) utilising canola oil methyl ester. They want to compare it to the output responses of a four-stroke four-cylinder turbocharged (TC) DI Land Rover 110 diesel engine's light absorption coefficient (LAC), NOx, O2, CO2, CO, EGT, BTE, BSFC, BMEP, braking power (Bp), and brake torque. Nine tests were scheduled to create the mathematical model for response prediction using central composite face centred design (CCFCD).

Singh et al. [86] conducted experiments with a four-stroke, single-cylinder DI diesel engine at a constant engine speed of 1500 rpm. The engine was operated at varying injection pressures (160, 180, 200, 220, and 240 bar), injection timing (15°, 19°, 23°, 27°, and 31° CA bTDC), and engine load using diesel fuel-cassia tora methyl ester mixes (0, 10, 20, 30, and 40 %). (20%, 40%, 60%, 80%, and 100%). The input components were related to the output responses BTE, HC, and NOx. The optimal combination was discovered at 47% engine load, 40% cassia tora with diesel, 221 bar fuel injection pressure, and 15° CA bTDC injection timing, according to the 31 trial runs employing the central composite rotating design. It produced the least amount of NOx and HC while increasing BTE.

Yilmaz et al. [87] investigated the effects of fuel injection timing (12°, 15°, and 18° CA bTDC) and engine speeds (2000, 3000, and 4000 rpm) on the performance and exhaust emissions of a Land Rover 110 turbocharged, DI, four-stroke, four-cylinder diesel engine using Hazelnut oil methyl ester. For this objective, RSM models were constructed for each response, including braking power, BSFC, BTE, EGT, CO, CO2, NOx, and smoke opacity. These findings show that regression models based on central composite face centred design efficiently model responses with high accuracy.

Khandal et al. [88] carried out an investigation using honge biodiesel in a Kirloskar single-cylinder, four-stroke, DI CI engine to analyse the effect of different EGR ratios (15%, 20%, 25%), fuel injection pressure (800, 900, and 1000 bar), and fuel injection timing (5°, 10°, and 15° CA bTDC) towards peak pressure (Pmax), heat release rate (HRR), ignition delay (ID), combustion duration (CD), BTE, CO, HC, NOx, and smoke opacity. The studies were carried out in 27 runs utilising a complete factorial design. The engine performance of the common rail DI (CRDI) diesel engine with 21 % EGR, 10° CA bTDC of injection timing, and 900 bar of fuel pressure was found to be better than the standard-setting diesel engine.

Kumar and Dinesha [89] studied the use of honge methyl ester blend fuels with diesel fuel by volume of 15 %, 20 %, and 25 % in single-cylinder, four-stroke, CI engines. The RSM model was created to examine the effect of varying compression ratios at (16, 17, and 18), injection timing at (24°, 27°, and 30° CA) bTDC, and engine load percentage at (50, 75, and 100%) load on the output response model of BTE and NOx. This research looked at 31 runs based on the central composite design with three-level components. It was observed that the optimal settings were 26.24° CA bTDC injection time, compression ratio of 16, 15 % honge methyl ester, and 86.3% engine load. NOx and BTE levels were 220 ppm and 31.5%, respectively.

Ganapathy et al. [90] tested jatropha biodiesel engine performance in a single-cylinder, DI 8.3 kW Greaves Cotton diesel engine. The study's goal was to offer a technique for the thermodynamic model analysis of a jatropha biodiesel engine in conjunction with Taguchi's design (TD) approach to discover the best engine design and operating parameters. The experiment was designed around 10 important operational parameters connected to BTE as output response. These 10 input characteristics were considered input factors, and each component was assigned two levels. The findings showed that the Taguchi TD approach-based thermodynamic model enhanced the performance parameters.

Using a four-stroke, single-cylinder, VCR CI engine, Patel et al. [91] investigated the optimal percentage of diesel fuel with Jatropha curcas bio-oil from the pyrolysis process at a ratio of 4%, 8%, 12%, and 16% by volume. In that study, the central composite design was used, with a total of 20 tests. Three independent factors were chosen: fuel blends, engine load (2 kg, 6 kg, and 10 kg), and compression ratio (14, 16, and 18), with HC, CO2, CO, BTE, and BSFC as output responses. It is worth noting that the best results were obtained with a mix of 12.22%, an engine load of 6.665 kg, and a compression ratio of 18.

It is critical to optimise those input elements aimed at lowering exhaust emissions and improving engine performance. Using a four-stroke, four-cylinder, DI diesel engine, Xu et al. [93] tested 20 % jatropha curcas biodiesel combined with 80 % diesel fuel. They discovered the impact of this fuel blend on engine performance and exhaust emissions with different pilot-main injection intervals (II) (0°, 5°, and 10° CA), start of injection timing (2°, 7°, and 12° CA) bTDC, fuel injection pressure (130, 145, and 160 MPa), and engine load (20 %, 60 %, and 100 %). The results of the four-factor, three-level complete factorial design showed that the optimal parameters were at the pilot-main injection interval of 4.40° CA, the start of injection timing of 4.02° CA bTDC, the fuel injection pressure of 160 MPa, and the engine load of 65.71%.

In another study, Xu et al. [93] employed a 20% mix of jatropha curcas biodiesel with diesel fuel in a four-cylinder water-cooled turbocharged diesel engine under light engine load operation. That research provided important insights into the optimization of engine performance and exhaust emissions characteristics models such as BSFC, BTE, HC, NOx, and soot. The fractional factorial design was used in this study's RSM approach. The input variables used for this study were pilot-main injection intervals (1°, 6°, and 11° CA), injection start time (1.5°, 6.5°, and 11.5° CA bTDC), and fuel injection pressure (110, 125, and 140 MPa). There are 80 experimental runs in the fractional factorial design analysis. The results showed that 5.8° CA pilot-main injection intervals, 6.4° CA bTDC start of injection timing, and 134.11 MPa fuel injection pressure were the best conditions for the fuel blend by 37.31%, 233.26 g/kWh, 12.73 ppm, 0.037 FSN, and 603.44 ppm, respectively, for BTE, BSFC, HC, soot, and NOx.

Dhingra et al. [94] investigated the optimization of combustion, performance, and emissions parameters in a four-stroke, single-cylinder, DI CI engine powered by jatropha curcas biodiesel-diesel fuel blends using the central composite rotatable design of RSM and non-dominated sorting genetic algorithm-II (NSGA-II). With a total of 20 trials, they adjust the compression ratio, engine load torque, and fuel blending ratio at five levels. The experimental investigation yielded the following results: smoke, NOx, HC, CO, BTE, BSFC, and peak cylinder pressure. The major goal of their research was to determine the optimal settings for higher brake thermal efficiency (BTE) performance and reduce Pmax, BSFC, CO, HC, NOx, and smoke based on the results of the RSM primary analysis. The best values were found in gasoline blends ranging from 17.35% to 22.63%, engine load torque ranging from 7.98 Nm to 11.62 Nm, and compression ratio ranging from 14.92 to 15.80.

Dhingra et al. [94] used the RSM approach to investigate a fatty acid ethyl ester from mahua oil combined with diesel fuel in a four-stroke, single-cylinder, variable compression ratio Kirloskar (model-AV1) diesel engine. As input factors, five variables were chosen: engine load, fuel mixes ratio, compression ratio, engine speed, and injection time. The central composite rotatable design was used in the investigation, which resulted in 32 experiment runs. Variations in peak in-cylinder pressure, BSFC, BTE, CO, HC, NOx, and smoke opacity were effectively investigated using RSM. The optimal compression ratio, engine speed, injection time, engine load, and blending ratio were determined to be 15.50:1, 2920 rpm, 345 CAD, 75 %, and 23 %, with a maximum desirability of 0.7346.

Pandian et al. [48] investigated the engine performance characteristics and exhaust emission in a twin-cylinder, normally aspirated, DI diesel engine utilising a three-factor fractional factorial design fueled with 40% Pongamia methyl esters and 60% diesel fuel mix. The nozzle trip protrusion (NTP), injection time, and injection pressure were selected as input components for the RSM model in this investigation. The nozzle trip protrusion varied at three stages, from 1 mm to 4 mm, with a 1.5 mm interval. The injection pressure changed in five stages of 25 bar, from 150 bar to 250 bar. The injection time was changed in five stages from 18° bTDC to 30° bTDC. The selected input elements were important in predicting the engine's performance metrics (BSEC and BTE) and emissions (CO, HC, NOx, and smoke opacity). The research recommended that nozzle tip protrusion of 2.5 mm, injection timing of 21° CA bTDC, and injection pressure of 225 bar were the best values for the Pongamia biodiesel-diesel fuel mix in the test engine when run at a constant speed of 1500 rpm.

Singh et al. [95] used central composite rotational design to determine the influence of the input parameters of engine load, Pongamia methyl esters blends (0%, 10%, 20%, 30%, and 40% by volume), fuel injection timing (15°, 19°, 23°, 27°, and 31° bTDC), and fuel injection pressure (160, 180, 200, 220, and 240 bar) and the output of the predicted model of BTE, HC and NOx of four-stroke, single-cylinder, DI diesel engine. With a high desirability optimization value of 0.967, it was determined that 53.13% engine load, 40% biodiesel mix, 196.36 bar fuel injection pressure, and 5° bTDC injection time were ideal for this engine. Furthermore, based on the aforesaid data, the engine's performance was judged to be greatly enhanced.

Najafi et al. [96] employed two kinds of methods to create one successful approach for forecasting and modelling goals: RSM and an artificial neural network model. A fatty acid ethyl ester of waste cooking oil combined with diesel fuel was tested in a Margo naturally aspirated, single-cylinder, four-stroke, CI engine in that research. Engine loads and fuel blends were varied at ten levels (10%, 20%, 30%, 40%, 50%, 60%, 70%, 80%, 90%, and 100% of full engine load) and eleven levels (0, 10%, 20%, 30%, 40%, 50%, 60%, 70%, 80%, 90%, and 100% of blended biodiesel-diesel fuel). The study's regression models focused on engine performance (exhaust gas temperature, exergy efficiency, Exergy, and energy efficiency, Energy). The optimum exergy and energy efficiency conditions might be observed in the range of 25 %–30 % of full engine load.

Shirnechan et al. [97] used a four-stroke, turbocharged CI engine (OM314) and waste cooking oil (WCO) biodiesel to forecast engine performance utilising the central composite rotatable design. Based on a three-factor five-level design, the major metrics for measuring the engine's performance were engine speeds, engine loads, and the biodiesel % in fuel combination (biodiesel and diesel fuel). The used design included 20 trials including output responses and input components. BSFC, braking torque, and brake power were the observed responses.

Hirkude and Padalkar [98] investigated the engine performance of a four-stroke, single-cylinder, DI diesel engine utilising waste cooking oil biodiesel. The researchers used RSM historical design data to do multi-objective optimization. A total of 36 runs were carried out with different injection timing (24°, 27°, and 30° bTDC), injection pressure (200, 225, and 250 bar), and compression ratio (16, 17, 18, and 19) as input parameters. Smoke opacity, EGT, BTE, and BSFC are the output response variables. The ideal compression ratio, injection pressure, and injection timing parameters observed via RSM were 17.99:1, 250 bar, and 27° bTDC, with output responses of 29.76% of BTE, 0.289 kg/kW h of BSFC, 298.52 °C of EGT, and 56.49 HSU of smoke opacity [99].

### 3.4 Additive and gaseous

Continuous NOx emission reduction, smoke opacity, and peak engine performance are the goals of the studies conducted by, Kumar et al. [99] using diesel fuel-oxygenate mixes. The oxygenated variety, delayed injection time, and oxygenated mixtures were the primary foci of this study. In this experiment, three common oxygenates—Dimethyl carbonate (DMC), Diethyl ether (DEE), and Diglyme (DGM)—were tested. These parameters have been adjusted from diesel fuel oxygenated blends proportions of 10 %, 15 %, and 20 % with delayed injection timings of 17°, 19°, and 21° CA. BSFC, BTE, CO, HC, NOx, and smoke opacity were the responses measured. Nine iterations involving the combination of output reactions and input components were conducted using the Grey-Taguchi method. When compared to reference diesel fuel, they showed that a 10 % DGM mix injected at 21° CA simultaneously achieved the greatest performance (7 %), reduced NOx emissions (17.4 %), and decreased smoke opacity (29.17 %) by the use of the desirability method of RSM.

Using a four-stroke, single-cylinder, normally aspirated, DI diesel engine, Kumar et al. [51] tested blending ratios of 38:62, 45:55, and 15:85 by volume for *n*-pentanol, iso-butanol, and dimethyl-carbonate, respectively, with diesel fuel. The injection time was varied by 21°, 23°, and 25° CA bTDC, while the EGR rate was varied by 0 %, 15 %, and 30 % in a three-factor-three-level complete factorial design. It was decided to measure the BSFC, CO, HC, NOx, and smoke opacity as the output responses. Based on the research conducted, it was determined that the most desirable operating condition involves an injection timing of 22° CA bTDC with an iso-butanol/diesel fuel mix and no exhaust gas recirculation (EGR).

Using an Ashok Leyland ALU WO4CT (four-stroke, direct-injection, turbocharged with intercooler) CI engine, Dhole et al. [108] studied the use of hydrogen (H2) gas with diesel fuel operation at different gaseous fuel substitution (0%, 5 %, 10 %, 15 %, 20 %, and 25 %) and engine load (13 %, 40 %, 60 %, and 80 %). In this study, we used a general factorial design (GFD)-based RSM tool to create four separate mathematical models for BTE, CO, HC, and NOx. The results of this study show that the constructed models are statistically significant at the 95 % level.

Dhole et al. [100] used the same methods as their prior work, but with a new dual-fuel combination (diesel fuel and producer gas from rice husk as secondary fuel) to analyse mathematical modelling of the experimental examination. Primary process variables developed to link with response variables like BTE, CO, HC, and NOx included engine loads (13 %, 40 %, 60 %, and 80 %) and gaseous fuel substitutes (0 %, 10 %, 20 %, 30 %, 40 %, and 50 %). Experiment findings shown that RSM based on general factorial design is a robust method for acquiring experimental design and statistical analysis for effective experimentation.

Using a four-stroke, single-cylinder, direct-injection diesel engine Hosmath et al. [102] performed experimental study on the effects of compressed natural gas as the inducted fuel and honge oil methyl ester (HOME) as the injected fuel. The engine is capable of using both gasoline and diesel fuel. The primary experiment was designed to determine the correlation between the output responses of smoke, CO, HC, NOx, ignition delay, combustion duration, BTE, rate of heat release, and peak in-cylinder pressure and the injection timing (19°, 23°, and 27° bTDC), CNG flow rate (0.25 kg/h), and compression ratio (0.50 kg/h), respectively. The results of their complete factorial design study showed that using CNG and HOME together increased BTE and decreased CO, HC, and smoke emissions.

### 3.5 Ternary fuels

In the literature on hybrid fuels, ternary fuel's benefits in lowering exhaust pollutants like PM and toxic gases have stood out [117]. However, the evidence linking the two is hazy at best [118], what with fuel efficiency and engine longevity both playing a role.

To predict the impact of using gasoline as the premixed fuel and diesel fuel-waste cooking oil (WCO) methyl ester in a single-cylinder homogeneous charge compression ignition (HCCI) engine, Leo, Sekar, and Arivazhagan [103] performed a multi-objective optimization. Based on the central composite design, three numerical input elements were chosen: the premixed fuel ratio, the fuel blend, and the engine load. Variations in premixed fuel ratio (0, 0.1, and 0.2), fuel blend (0, 50, and 100%), and engine load (0, 50, and 100%) were used. The output responses that included BTE and exhaust emissions of CO, HC, NOx, and smoke were chosen. A rotational velocity of 1500 rpm was maintained throughout all the tests. The analysis concluded that a mixture of 48.0524% engine load, 100% WCO biodiesel, and zero premixed fuel ratio of gasoline fuel was the best possible combination, with a maximum attractiveness of 0.7771.

The impact of waste cooking oil and ethanol mix levels, operational variables, engine speed, and engine load on the exhaust emissions of a four-cylinder, compression ignition diesel engine was studied by Khoobbakht et al. [104]. Blending ratios of 0, 0.1, 0.2, 0.3, and 0.4 ethanol to diesel fuel or biodiesel to diesel fuel were used (0, 0.2, 0.4, 0.6, and 0.8). The engine speed was changed from 1000 to 1450 to 1900 to 2350–2800 rpm, and the engine load changed from 20 % to 60 %–80 % to 100 %. Response parameters including CO, CO2, HC, NOx, and smoke opacity might be predicted with the use of experiments based on the central composite rotatable design (CCRD) of RSM. Based on the examination, it was determined that a combination of 63 % diesel fuel, 11 % ethanol, and 26 % biodiesel produced the best results for the test engine at a speed of 2800 rpm with 80 % of maximum engine load.

Blends of diesel fuel, ethanol, and soybean oil methyl ester were investigated by Khoobbakht et al. [105]. The impact of operational factors on the output response parameter of brake exergy efficiency was studied. These parameters included engine speed and engine load (BEE). The ratio of biodiesel to diesel fuel was varied over five levels (0, 0.2, 0.4, 0.6, and 0.8), the ratio of ethanol to diesel fuel was varied over five levels (0.0, 0.1, 0.2, 0.3, and 0.4), and the speed of the engine was varied over five levels (1000 rpm, 1450 rpm, 1900 rpm, 2350 rpm, and 2800 rpm): engine stress (20%, 40%, 60%, 80%, and 100%). Researchers found that the optimum conditions for BEE (36.72 %) occurred at 94 % engine load, 1900 rpm engine speed, 17 % biodiesel with 8 % ethanol added to diesel fuel, and 100 % ethanol.

Using a four-stroke, four-cylinder, turbocharged Land Rover 110 diesel engine, Atmanli et al. [106] conducted three optimization tests on ternary mixes of cotton oil, butanol, and diesel fuel. The input variable was chosen to be a fuel mixing ratio with seven distinct concentrations. Engine performance data including BMEP, BTE, BSFC, max braking power, max brake torque, and exhaust emissions like CO, HC, and NOx were also included in the replies. Using the RSM, a fuel mixture of 11.4 % cotton oil, 23.1 % butanol, and 65.5 % diesel fuel was found to provide ideal conditions with a maximum desirability of 0.83513. The similar result was found by Atmanli et al. [79] in a different study, but with a more ad hoc choice of experimental design components.

Many different types of input factors have been used by many different studies. In their study, Krishnamoorthy et al. [108] combined diesel fuel and WCO with a ternary mix of three bio-alcohols: *n*-pentanol, n-butanol, and n-propanol. RSM's 3-factor x 3-level complete factorial experimental design led to the selection of three input factors: EGR rates (10, 20, and 30%), injection time (23°, 25°, and 27° bTDC), and alcohols. Regression models were built using BSFC, BTE, CO, HC, NOx, and smoke opacity. Using 15 % EGR, 23° CA bTDC injection time, and a ternary mix of 20 % *n*-pentanol, 30 % WCO, and 50 % diesel fuel, we were able to get a maximum desirability of 0.974.

Using a Kirloskar single-cylinder, four-stroke, VCR test engine, Ramakrishnan, Kasimani, and Peer [109] gathered data. Three different engine loads (1.07 BMEP, 2.15 BMEP, and 4.16 BMEP) and three different compression ratios were used while the engine operated at a constant speed of 1500 rpm on diesel-pentanol- calophyllum inophyllum biodiesel blends (16:1, 17:1, and 18:1). BSFC, BTE, CO, CO2, HC, NOx, and smoke were selected as inputs to correspond with the aforementioned responses. Based on experimental results from a complete factorial design, the optimal values for the input parameters—engine load of 2.5 BMEP, compression ratio of 17.5:1, and fuel mix of 20 % biodiesel, 20 % pentanol, and 60 % diesel fuel—are both 0.88.

Using a four-stroke, single-cylinder, variable compression ratio, and multi-fuel CI research engine, Krishnamoorthi et al. [110,111] assessed tri-fuel blends of diesel fuel, diethyl ether, and Aegle marmelos (bael) plain oil. Injecting at 21, 23, and 25° before top dead centre (bTDC), injecting at 210, 230, and 250 bars of pressure, and varying the compression ratio from 16:1 to 18:1 were all factors in the RSM factorial design (FD). The study found that the three various types of test fuel all showed somewhat different optimal settings, with maximum composite desirability ranging from 0.5683 to 0.6168, under the experimental condition of 1500 rpm (or 25 rps) and 80 % engine load. The input parameters were also shown to significantly affect smoke opacity, NOx, HC, CO, BTE, and BSFC, according to the study's findings.

Another study looked at the performance of diesel fuel, diethyl ether, and Choulmoogra neat oil (65 % diesel fuel, 10 % diethyl ether, and 25 % Choulmoogra oil) in a single-cylinder, four-stroke, VCR, CI engine by Krishnamoorthi and Malayalamurthi [112]. In this investigation, we used the RSM strategy predicated on a three-factor and four-level factorial layout. Smoke, NOx, HC, CO, BTE, and BSFC were chosen as output responses, with engine speed (1500 rpm, 1800 rpm, 2100 rpm, and 2400 rpm), compression ratio (14.5:1, 16:1, 18.1:1, and 20.6:1), and EGR (0 %, 10 %, 20 %, 30 %) serving as input factors. It's worth noting that a composite desirability of 0.665 was achieved at an engine speed of 1672 rpm, 10 % EGR, and 18.1 CR to achieve the optimal conditions.

Krishnamoorthi et al. [113] conducted an additional study using the same fuel mixture and experimental setup, determining that an EGR rate of 0 %, 5 %, and 10 % resulted in the lowest emissions and highest performance. The inputs to this study were engine speed, engine load, and compression ratio, while the output variables were carbon monoxide, carbon dioxide, hydrocarbon, nitrogen monoxide, smoke, benzene, toluene, and ethyl benzene. Experiments were conducted with compression ratios of 17:1, 18.1:1, 19.2:1, and 20.6:1, and with engine speeds of 1500, 1800, 2100, and 2400 revolutions per minute. The results of the factorial design indicated that the optimal composite desirability is within the range 0.593–0.628.

In a series of tests utilising a single-cylinder, four-stroke CI engine, Patel, Lakdawala, and Patel [114] combined 76% diesel fuel with 4% diethyl ether and 20% biodiesel. In this study, the RSM Box-Behnken layout was used, and the injection time, injection pressure, and compression ratio were the input variables. There were 15 separate experiments used in this Box-Behnken analysis. Insights regarding how to best optimise output responses were provided by the research (BTE). Finally, a compression ratio of 18:1, injection pressure of 220 bar, and injection timing of 20° bTDC were determined to be the ideal conditions for the engine.

Using a test engine with a tunable compression ratio, Shameer and Ramesh [50] studied the effects of a mixture of various antioxidants including 20 % *Calophyllum inophyllum* (CI) methyl ester. Compression ratios of 16.5, 17.5, and 18 and injection times of 21°, 22°, 23°, and 24° CA before peak cylinder filling (bTDC) were tested. Based on the results of the experiments, the following answers were chosen as possible outputs: BSFC, BTE, CO, CO2, HC, NOx, and smoke opacity. The study's primary objective was to determine the antioxidant/biodiesel blend's sweet spot in terms of reduced emissions and enhanced engine performance. Injection timing of 23° bTDC, compression ratio of 17.5, and fuel mix containing 1000 ppm THBQ were determined to be the best parameters via a complete factorial design study of RSM.

Using a biodiesel/diesel fuel combination in a single-cylinder, four-stroke, water-cooled Kubota RK-125 diesel engine, Wu and Wu [115] explored the introduction of hydrogen and cooled EGR at intake port. The optimal solution is determined by selecting three input factors: the percentage of EGR, the ratio of hydrogen energy sharing, and the mix of biodiesel and diesel fuel. Twenty, thirty, and forty percent EGR were tested. The volume ratio of biodiesel to diesel fuel was changed from 10 to 20 to 30%, while the energy-to-hydrogen-share ratio was changed from 10 to 20 to 30%. Based on the Taguchi method's optimum combination findings, the researchers found that a 40 % EGR ratio, 30 % hydrogen, and a 20 % biodiesel mix provided the best BSFC, BTE, NOx, and smoke across all engine loads.

Using a four-stroke, single-cylinder CI engine, Bose et al. [116] investigated the impact of fuel mixes including diesel fuel, ethanol, hexane, and diethyl ether (as an ignition improver). Using the volumetric percentages of the fuel mix components and the engine load as the input parameters, the D-optimal design of RSM was evaluated. When testing the output response parameters of NOx, HC, CO, and BSFC, we subjected the engine to loads of 2, 2.5, 3, 3, and 4 kg (50, 75, 95, and 100 % of full load, respectively). Multi-optimization using RSM proved to have produced a 40 % diesel fuel, 40 % ethanol, 15 % DEE, and 5 % hexane input condition at 95 % full engine load with less than 18 % absolute error compared to real experimental values.

## 4 Improving RSM

Although RSM comes with many benefits compared to other optimization methods, it also has some drawbacks, as summarized in Table 3. One of the most critical matters to be tackled when utilising RSM is the initial position. Frequently, the first estimation of input factors will not accurately be near the actual optimum point. Afterwards, the researcher must shift to the optimum area using the steepest descent or ascent method to produce the most effective integration of factors [119].

**Table 3:** Benefits and drawbacks of RSM [55,[119], [120], [121]].

Benefits
Produce a massive quantity of data from a slight amount of trials.
The critical influence of factors and interaction influence among the factors on the output responses can be analyzed.
The model generated can forecast output response.
Visual diagrams aids in the graphical understanding of the valuable connection between the input factor and the output response.
Discover the level of factor which offer the optimal response.
Many responses can be studied, and the optimal parameters considering the whole responses can be discovered.
As the trials are carefully designed, the period of the study can be predicted.
The response responsiveness to the related input factors is possible to be evaluated.
**Drawbacks**
The number of trials becomes more significant with the increasing amount of input factors.
Low forecast quality for outside the investigational area.
The investigational trial data is suitable solely for first or second-order polynomials.
The input factors selected must be continuously different throughout the investigational area.
Model possible to explain the situation that occurs at various situations. However, unable to clarify the natural phenomena of the operation.

Another drawback is the limited experimental area, as RSM cannot develop a model over a broad area. Therefore, the researcher must understand the process being investigated to choose the experimental area [120]. Besides, RSM cannot explain all curvature systems as second-order polynomial usually used for modelling [121].

The RSM's drawbacks can be reduced by merging with other optimization methods. One of the methods is the artificial neural network (ANN), which offers nonlinear modelling for response surfaces. ANN utilizes all points from the experimental data to predict the output response. Analysis using ANN usually is flexible for the form and number of the experimental data. Furthermore, merging ANN with RSM would provide an excellent data learning and prediction than the standard RSM. On the other hand, the ANN method requires many experiments compared to RSM [122].

The combination of RSM with other modelling approaches such as genetic algorithm [123], simulation annealing [124], and fuzzy logic [125] had also been explored. These approaches had been verified to enhance the RSM to discover the optimum close points. Even though this method had been implemented in some other disciplines, a small number of investigations have been reported on their implementation in analyzing/optimizing engine combustion, performance, and exhaust emissions.

Another potential improvement in RSM is by integrating it with a uniform design approach. By applying a uniform design approach, the number of investigation trials is determined solely by the factor levels, not through the number of factors. Therefore, this approach is suitable for utilization, especially when the factor level is not the same. Moreover, the combined method can optimise the process using less experiment and would be attractive for a condition where the running process is expensive [126].

## 5 Summary

This article has delved into diverse studies focusing on the combustion, performance, and exhaust emissions of Internal Combustion Engines (ICE) tested through the Response Surface Method (RSM). The results reveal that RSM is adept at accurately predicting the relationship between input factors and output responses across various engine tests, particularly those involving engine parameters and alternative fuels in ICE. Consequently, it is reasonable to assert that RSM holds specific advantages and limitations depending on its application.

A thorough examination of the existing literature suggests that RSM plays a pivotal role in optimizing engine combustion, performance, and emissions, especially when varying engine parameters and alternative fuels are involved. The method aids in identifying the optimal parameters among a spectrum of input factors and output response variables. Implementing this strategy necessitates choosing an appropriate experimental design, fitting a suitable mathematical function, and scrutinizing the quality and accuracy of the fitted model to generate predictions aligned with the collected experimental data. The pattern of relationships between output responses and input factors is intrinsically linked to the statistical results derived from the chosen design.

In certain instances, the coefficient of determination for RSM may be notably low, indicating a limitation in the predictive model. This constraint can be mitigated by adjusting the weights assigned, based on the significance of the output response parameters. The review outlined in this article indicates that central composite design and factorial design are prevalently employed. However, the utilization of other designs such as Box-Behnken, user-defined, historical data, Taguchi, orthogonal experimental, and D-optimal designs is less commonly documented and investigated.

Given that ICE operation is influenced by a multitude of uncontrollable variables, understanding the relationship between input factor parameters and the output responses of ICE is crucial. Therefore, employing RSM as a predictive tool is vital for enhancing the precision in determining the significance of operating parameters in ICE. Moreover, integrating RSM with alternative optimization techniques is likely to yield greater accuracy in data learning and estimation compared to using RSM alone.

## Data availability statement

No. No data was used for the research described in the article.

## Additional information

No additional information is available for this paper.

## CRediT authorship contribution statement

**Johnny Koh Siaw Paw:** Investigation, Funding acquisition. **Tiong Sieh Kiong:** Supervision, Investigation. **Mohd Kamal Kamarulzaman:** Writing – original draft, Methodology. **Abdullah Adam:** Visualization, Supervision. **Sakinah Hisham:** Writing – original draft, Project administration. **K. Kadirgama:** Writing – review & editing, Writing – original draft. **D. Ramasamy:** Methodology. **Chong Tak Yaw:** Writing – review & editing, Formal analysis. **Ahmad Fitri Yusop:** Writing – original draft, Investigation. **Talal Yusaf:** Project administration, Funding acquisition, Formal analysis. **Hayder A. Dhahad:** Supervision, Methodology. **F. Benedict:** Investigation, Conceptualization.

## Declaration of Competing Interest

The authors declare that they have no known competing financial interests or personal relationships that could have appeared to influence the work reported in this paper.

## Nomenclature

**Abbreviations**
AC: Air-cooled
ANN: Artificial neural network
aTDC: After top dead centre
BBD: Box-Behnken design
Bd: Biodiesel
BEE: Brake exergy efficiency
BHA: Butylated hydroxyanisole
BHT: Butylated hydroxytoluene
BMEP: Brake mean effective pressure
BP: Boost pressure
Bp: Brake power
BSFC: Brake specific fuel consumption
bTDC: Before top dead centre
BTE: Brake thermal efficiency
Bu: Butanol
CA: Crank angle
CCD: Central composite design
CCFCD: Central composite face centred design
CCRD: Central composite rotatable design
CD: Combustion duration
CI: Compression ignition
CR: Compression ratio
CRDI: Common rail direct injection
CNG: Compress natural gas
DEE: Diethyl ether
DF: Diesel fuel
DGM: Diglyme
DI: Direct-injection
DMC: Dimethyl-carbonate
DO: D-optimal
DOE: Design of experiments
Dwell: Time interval between two diesel injections
EGR: Exhaust gas recirculation
EGT: Exhaust gas temperature
EL: Engine load
ES: Engine speed
Eth: Ethanol
FAEE: Fatty acid ethyl ester
FAME: Fatty acid methyl ester
FAWE: Fusel oil after water extraction
FB: Fuel blend/ratio
FCE: Fuel conversion efficiency
FD: Factorial design
FEF: Fumigant energy fraction
FFD: Full factorial design
FIP: Fuel injection pressure
FR: Fuel/flow rate
FrFD: Fractional factorial design
FT: Fuel type
GF: Gasoline fuel
GFD: General factorial design
HCCI: Injection homogeneous charge compression ignition
HDD: Historical data design
Hex: Hexane
HRR: Heat release rate
HSDI: High-speed direct-injection
IC: Intercooler
ID: Ignition delay
IDI: Indirect injection
II: Injection interval
IMEP: Indicated mean effective pressure
Inj1Fr: Fraction of first diesel injection
IP: Injection pressure
IT: Injection timing
LAC: Light absorption coefficient
MK: Modulated kinetics
NA: Natural aspirated
NG: Natural gas
NSGA: Non-dominated sorting genetic algorithm
NTP: Nozzle tip protrusion
Oct: Octanol
Pe: Pentanol
PG: Propyl gallate
P_int_: Intake air pressure
PM: Particulate matter
Pmax: Peak in-cylinder pressure
Pr: Propanol
P_rail_: Diesel fuel rail injection
PY: Pyrogallol
RCCI: Reactivity-controlled compression ignition
RSM: Response surface methodology
SI: Spark ignition
SOCH: Single overhead camshaft
SOI: Start of injection
SOIC2: Start of injection command for the 2nd injection
TC: Turbocharged
TD: Taguchi design
T_int_: Intake air temperature
UDD: User-defined design
VCR: Variable compression ratio
WC: Water-cooled
WCO: Waste cooking oil
WOT: Wide-open throttle
2D: Two-dimensional
3D: Three-dimensional
3^3^: 3 factor × 3 level
4 S: Four-stroke
η_mech_: Mechanical efficiency
η_Energy_: Energy efficiency
η_Exergy_: Exergy efficiency

**Chemical symbols**
CH_4_: Methane
CO: Carbon monoxide
CO_2_: Carbon dioxide
HC: Hydrocarbon
H: Hydrogen
NO_X_: Nitrogen oxides

**Mathematical symbols**
*β*_*0*_: Regression coefficient for the intercept
*β*_*i*_: Regression coefficients of the linear parameters
*β*_*ii*_: Regression coefficients of the quadratic parameter
*β*_*ij*_: Regression coefficients of the interaction parameters
*c*_*p*_: Number of duplicates at the central point
D: Overall desirability function
*d*_*i*_: Value specifies the response value Y_i_ to be more desirable
*ε*: Residual (error) associated with the experiments
*f*: Number of distinctly different factor combinations
*f(x)*: Vector function of p elements
*High*: Upper limit of the response
*k*: Number of input factors
*Low*: Lower limit of the response
*n*: Number experiments in the set
*p*: Number of parameters in the model
R: Importance
*SS*_*Res*_: Sum square of residual (error)
*SS*_*T*_: Total sum of squares
*T*: Target value of the response
*wt*: Weight of the response
*x*_*0*_: Uncoded *i*th independent variable at the centre point
*x*_*i*_: Dimensionless coded value of the independent variable
*Y*_*i*_: Estimated response
y_i_: Observed value
ŷ_i_: Predicted value
*z*_*i*_: Uncoded value of the *i*th independent variable
*Δz*_*i*_: Step change value between the low level (−1) and high level (+1)
